# Antibody-Negative Paraneoplastic Autoimmune Multiorgan Syndrome (PAMS) in a Patient with Follicular Lymphoma Accompanied by an Excess of Peripheral Blood CD8+ Lymphocytes

**DOI:** 10.3390/curroncol29040194

**Published:** 2022-03-28

**Authors:** Thilo Gambichler, Yi-Pei Lee, Ilske Oschlies, Christina H. Scheel, Wolfram Klapper, Nico Nowack, Martin Doerler, Markus Stücker, Nasreddin Abolmaali, Laura Susok

**Affiliations:** 1 Skin Cancer Center, Department of Dermatology, Ruhr-University Bochum, 44791 Bochum, Germany; yi-pei.lee@klinikum-bochum.de (Y.-P.L.); christina.scheel@klinikum-bochum.de (C.H.S.); nico.nowack@klinikum-bochum.de (N.N.); m.doerler@klinikum-bochum.de (M.D.); m.stuecker@klinikum-bochum.de (M.S.); l.susok@klinikum-bochum.de (L.S.); 2 Hematopathology Section and Lymph Node Registry, Institute of Pathology, University Hospital Schleswig-Holstein, Campus Kiel, Christian-Albrechts University, 24105 Kiel, Germany; ioschlies@path.uni-kiel.de (I.O.); wolfram.klapper@uksh.de (W.K.); 3 Institute for Diagnostic and Interventional Radiology and Nuclear Medicine, Ruhr-University Bochum, 44791 Bochum, Germany; nasreddin.abolmaali@kklbo.de

**Keywords:** paraneoplastic pemphigus, autoimmune blistering diseases, cancer, autoantibodies, cytotoxic lymphocytes

## Abstract

Paraneoplastic autoimmune multiorgan syndrome (PAMS) is a life-threatening autoimmune disease associated with malignancies. Here, we present a patient initially misdiagnosed with “chronic” Stevens–Johnson syndrome. Over a year later, the patient was diagnosed with stage IV follicular lymphoma and treated with an anti-CD20 antibody. At this time, his skin condition had significantly worsened, with erythroderma and massive mucosal involvement, including in the mouth, nose, eyes, and genital region. Histopathology revealed lichenoid infiltrates with interface dermatitis, dyskeratoses, necrotic keratinocytes, and a dense CD8+ infiltrate with strong epidermotropism. Direct and indirect immunofluorescence tests for autoantibodies were negative. Remarkably, we retrospectively discovered a chronic increase in peripheral CD8+ lymphocytes, persisting for over a year. Consequently, the patient was diagnosed with antibody-negative PAMS. Three weeks later, he succumbed to respiratory failure. This dramatic case highlights the challenges in diagnosing PAMS, particularly in cases where immunofluorescence assays are negative. Importantly, we observed, for the first time, a chronic excess of CD8+ peripheral blood lymphocytes, associated with PAMS, consistent with the systemic, autoreactive T-cell-driven processes that characterize this condition.

## 1. Introduction

Paraneoplastic pemphigus (PNP) was first described by Anhalt in 1990 [1]. PNP is a rare life-threatening autoimmune disease associated with neoplasia. Common to all PNP cases is the presence of extensive and frequently severe lesions of mucosal membranes, including oral, conjunctival, nasal, and genital. Indeed, mucosal lesions often represent the earliest clinical symptoms of PNP [1,2,3,4,5]. Eye involvement is observed in about 70% of PNP patients, and can result in an irreversible loss of vision. Internal organ manifestations are frequent, particularly affecting the lungs and gastrointestinal tract. Notably, the incidence of lung involvement, usually presenting as bronchiolitis obliterans, ranges from 60% to 90% of PNP cases [1,2,3,4,5].

In 2001, Nguyen et al. introduced the concept of paraneoplastic autoimmune multiorgan syndrome (PAMS), focusing on systemic involvement [2]. PAMS has a mortality rate ranging from 50% to 90%, depending on the country reporting it [1,2,3,4,5,6]. Mortality is mainly determined by the prognosis of the underlying malignancy, and by frequently occurring lung complications, such as bronchiolitis obliterans and organizing pneumonia. Thereby, PAMS is clearly distinguished from classical pemphigus, which occasionally exhibits mucosal involvement of the pharynx and larynx. By contrast, pulmonary involvement, often severe enough to result in death, is never observed in classical pemphigus, but occurs in nearly all patients with PAMS [2].

PAMS usually presents with tender mucosal erosions, crusts, and dark-hued skin patches that subsequently desquamate. However, a great variety of skin manifestations, ranging from flaccid bullae to widespread lichenoid eruptions, have been observed in PAMS patients. Clinically, as well as histologically, PAMS can also mimic erythema multiforme, Stevens–Johnson syndrome (SJS), and toxic epidermal necrolysis (TEN), particularly when cutaneous lesions are characterized by interface dermatitis with necrotic keratinocytes [1,2,3,4,5,6,7]. Lymphoproliferative diseases represent the most frequently identified neoplasia associated with PAMS, accounting for over 80% of cases [1,2,3,4,5]. Almost 60% of PNP cases are associated with non-Hodgkin lymphomas and chronic lymphocytic leukemia. In fact, there are 21 cases of patients who suffered from follicular lymphoma (FL) associated with PAMS [5,6,7]. Among a broad range of possible proteins targeted by autoantibodies, plakin proteins (envoplakin, epiplakin, and periplakin) are the most characteristic of PAMS.

Here, we describe a patient who was initially diagnosed with vaccine-induced chronically recurrent SJS, accompanied by a chronic excess of peripheral CD8+ lymphocytes. One and a half years later, following the detection of follicular lymphoma, the patient was finally diagnosed with antibody-negative PAMS, with a fatal outcome three weeks later [7,8,9,10,11].

## 2. Case Report

**Figure 1:**
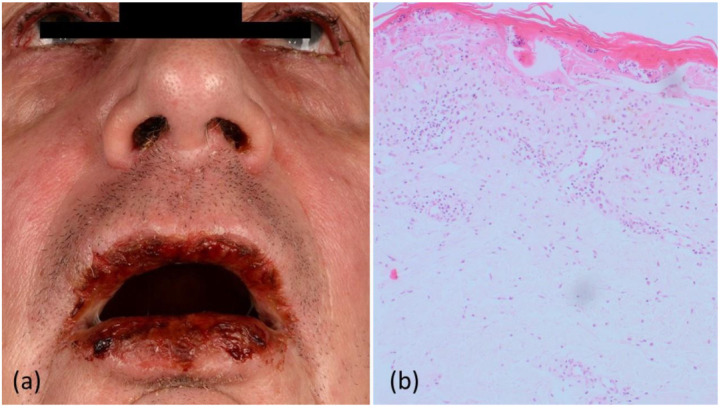
(**a**) Blepharo-conjunctivitis, together with bloody and crusty erosions on the lips and nostrils, (**b**) a punch biopsy of a macular lesion on the left arm, showing vacuolar interface dermatitis with focal necrotic alteration of the epidermis and an abundance of dyskeratoses. Of note, acantholysis was not observed.

A 70-year-old German male presented with a history of blistering oral lesions, which first developed in June 2020, one week following a vaccination against herpes zoster, pneumococcus, and rubella. In July 2020, he developed bloody and crusty erosions in the mouth, nasal mucosa, and conjunctiva (Figure 1). Moreover, erythematous papules and plaques were observed on his trunk and arms. The pathological laboratory parameters included leukocytosis, elevated C-reactive protein, and a low peripheral blood CD4/CD8 ratio of 0.79 (normal range: 0.8–2). Serology for bacterial and viral infections, including *Mycoplasma pneumonia* and HTLV1/2, was negative.

A histological analysis of a lesion biopsied from the left arm revealed partly lichenoid, vacuolized interface dermatitis, with an abundance of dyskeratoses and necrotic keratinocytes, but an absence of acantholysis (Figure 1). Of note, the patient had been on regular medication for arterial hypertension for many years, with no changes or new medication introduced recently. Subsequently, the patient was diagnosed with vaccine-induced SJS and treated with a tapered regimen of intravenous corticosteroids (2 mg/kg body weight), resulting in satisfactory clinical improvement.

**Figure 2:**
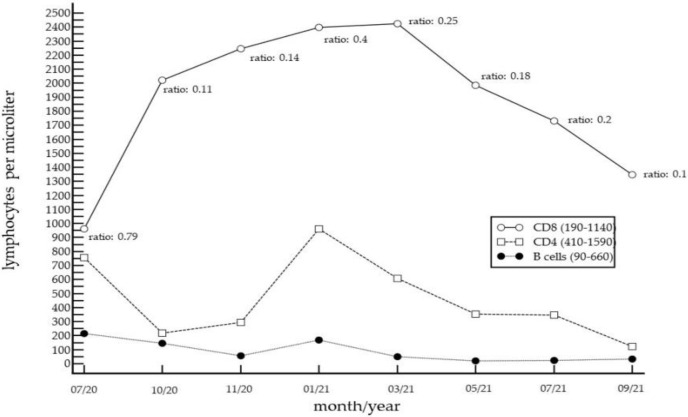
Timeline of peripheral lymphocyte subpopulation counts, from July 2020 to September 2021. The CD4/CD8 ratio (normal range: 0.8–2) is shown underneath the graph for CD8 T cells. Before PAMS developed, the ratio was close to normal (0.79 in 07/20), and then remained consistently low due to highly increased CD8+ lymphocyte counts. Of note, CD20+ B-cell counts were also low over most of the time.

Over the next 14 months, however, the initial symptoms recurred periodically, in particular, after cessation of oral corticosteroids and intravenously applied immunoglobulins. Over this period, the patient also experienced weight loss of about 20 kg. Since pathological findings were consistently detected in the patient’s peripheral lymphocyte subpopulations (Figure 2), laboratory tests for infectious agents were performed (e.g., for EBV, CMV, HSV, VZV, HTLV1/2, SARS-CoV-2, chlamydia, mycoplasma, and brucella). However, all the tests were negative. Blood smears, T-cell receptor rearrangement studies, and bone marrow examinations did not reveal evidence for conditions such as Felty syndrome, large granular lymphocytic leukemia, or other hematological conditions. Computed tomography of the chest excluded Good’s syndrome.

**Figure 3:**
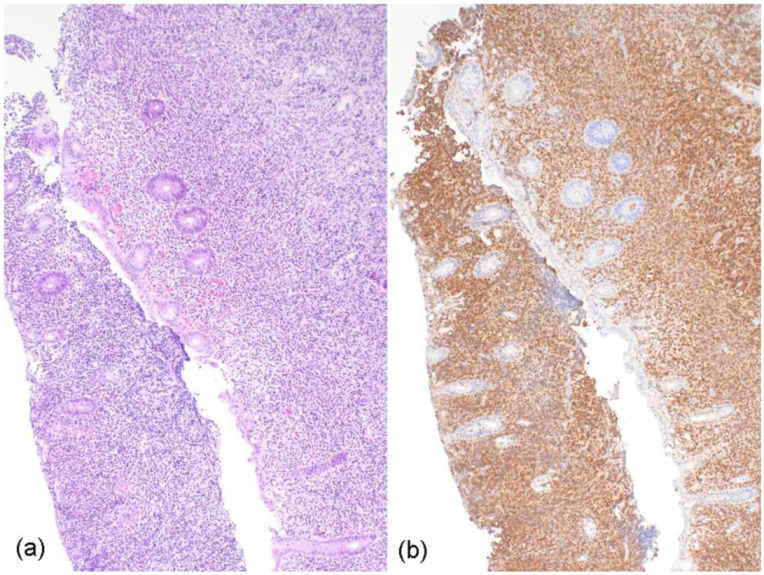
(**a**) Hematoxylin and eosin staining (10× magnification) of colonic mucosa, showing rarefied crypts, extensive erosions, and a dense and diffuse infiltration of the lamina propria by lymphocytes. (**b**) Immunohistochemistry (10× magnification) for CD20 shows strong staining in the diffusely infiltrating lymphoid cells. These neoplastic B cells were also positive for germinal center markers, such as CD10 and BCL6.

In September 2021, the patient complained of abdominal pain and blood in his stool. Colonoscopy revealed unremarkable rectum mucosa. By contrast, continuously morbid colonic mucosa was detected, extending over 18 cm from the proximal rectum. An ulcerative colitis-like pathology was suspected on the basis of diffuse hyperemic and edematous changes, friability, and several fibrinous ulcerations, with exudates in the mucosa. However, two biopsies of the colonic mucosa revealed infiltrates of grade I–II follicular lymphoma, which were strongly positive for CD20, Bcl-6, and CD10, as well as Bcl-2, with a Ki-67-idex of 15–20% (Figure 3); of note, no staining for cyclin-D1 was detected. FISH revealed *BCL2* translocation (18q21), but no *BCL6* translocation. Computed tomography staging showed para-aortal lymphadenopathy, with diffuse lymphatic tissue surrounding the left ureter, and sigmoid, as well as rectal, lymphatic infiltrates. Conclusively, the patient was diagnosed with stage IV follicular lymphoma (Ann-Arbor). A flow cytometry analysis of the peripheral blood, as well as an examination of a bone marrow sample, were unremarkable. Immuno-chemotherapy was initiated at the end of September 2021, with obinutuzumab (starting dose of 100 mg absolute) and bendamustine (125 mg on day 1 and 2). A second administration of obinutuzumab was carried out, with a dose of 1000 mg.

**Figure 4:**
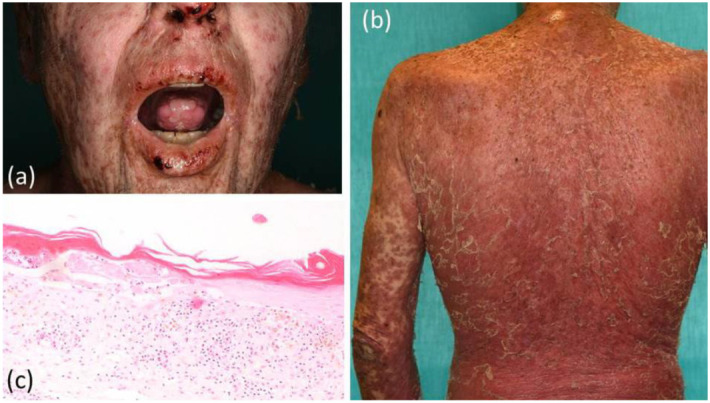
(**a**) Severe enoral blistering, together with bloody and crusty erosions on the lips and nostrils; (**b**) erythroderma covering over 80% of the patient’s body surface; (**c**) punch biopsy taken from a macular lesion from the left arm revealed lichenoid vacuolar interface dermatitis with focal necrotic alteration of the epidermis and abundance of dyskeratoses.

**Figure 5:**
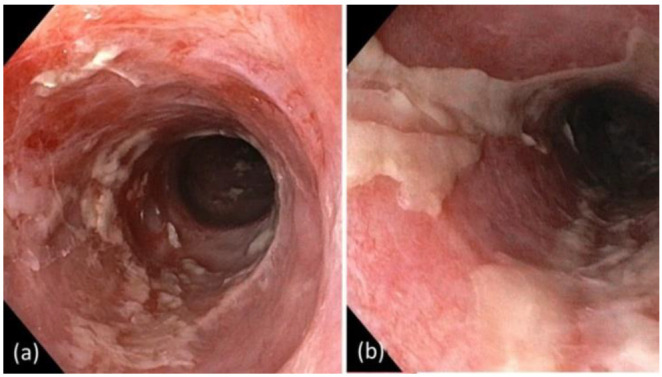
(**a**) Endoscopy of the esophagus revealing severe inflammation and contact-vulnerable mucosa along the entire esophagus, accompanied by erosions and massive fibrinoid deposits (**b**). Because of the marked mucosal vulnerability, biopsies were not performed.

The patient presented to our team for the first time in mid-October 2021. At this time, we observed scaly, erosive erythroderma over more than 80% of his body surface, accompanied by severe mucosal involvement, including in the mouth, eyes, nose, and genital region (Figure 4). Moreover, the patient had experienced almost complete loss of vision, due to severe chronic bilateral cicatrizing blepharo-conjunctivitis. Endoscopy revealed extensive inflammatory lesions and marked fibrinoid deposits along the entire esophagus (Figure 5).

**Figure 6:**
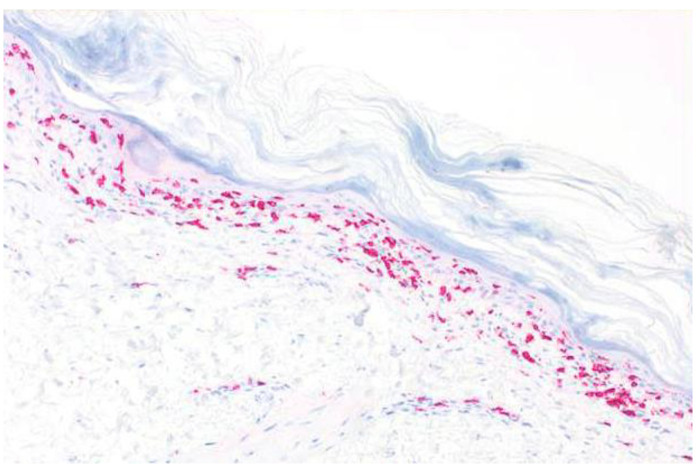
Immunohistology showing strong band-like immunoreactivity for CD8+ lymphocytes along the dermoepidermal junction zone, with strong epidermotropism.

A skin biopsy revealed a similar histopathological pattern as that observed in July 2020. Immunohistochemistry for cytotoxic lymphocytes revealed an abundance of CD8+ cells along the dermoepidermal junction zone, with marked epidermotropism (Figure 6). T-cell receptor rearrangement studies did not reveal evidence for T-cell lymphomas, such as primary cutaneous CD8+ aggressive epidermotropic cytotoxic T-cell lymphoma. In order to confirm PAMS, we carried out several laboratory tests. However, direct and indirect immunofluorescence studies did not reveal IgG or IgA autoantibodies. The ELISA for circulating BP-230, desmoglein 1 and 3, and envoplakin autoantibodies was negative.

**Figure 7:**
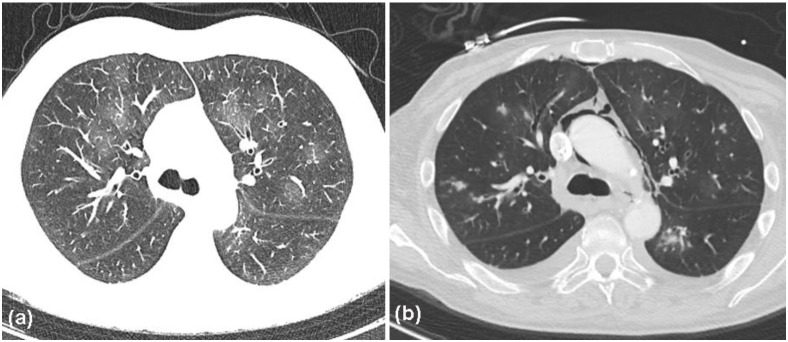
Computed tomography (CT) scans of the chest. (**a**) Subtle mosaic attenuation, ground-glass opacities, bronchiectases, and bronchial wall thickening, consistent with a diagnosis of bronchiolitis obliterans (BO); (**b**) pneumomediastinum as a further complication of BO.

Based on the overall clinicopathological findings and course of the disease, we were convinced that the patient did not suffer from chronic vaccine-induced SJS, but much more likely from antibody-negative PAMS, possibly due to the recent anti-CD20 therapy and triggering of massive auto-reactive T-cell responses. Notably, after the initiation of immuno-chemotherapy, the median absolute lymphocyte count from the end of September 2021 to the beginning of November 2021 was low, at 170/µL (normal range 100–740/µL, median of 12 assessments). These data suggested that B cells and the associated immunoglobulin production were massively suppressed by anti-CD20 treatment. Unfortunately, differentiation of the lymphocyte subpopulations was not performed during this period. However, because of the massive mucocutaneous involvement, high-dose intravenous corticosteroid therapy was administered (500 mg of prednisolone per day). Two days later, the patient complained of dyspnea, accompanied by elevated infection parameters (Table 1). Antibiotic therapy was initiated with ceftriaxone (2 g per day), and, after 3 days, was escalated to piperacillin/tazobactam (4.5 g, four times per day). Nonetheless, the patient developed progressive respiratory failure. At the end of October 2021, the patient was admitted to the intensive care unit. At this time, CT studies revealed that the extent of follicular lymphoma lesions remained more or less unchanged compared to previous imaging in September 2021. Endoscopic investigations showed severe sloughing of the esophageal epithelium. Furthermore, bronchoscopy revealed erythematous, highly vulnerable airway mucosa covered by purulent fluid. Several bronchoalveolar lavages revealed Pseudomonas aeruginosa, cytomegalovirus, Aspergillus fumigatus, and Candida albicans. Thoracic CT also revealed evidence for changes consistent with bronchiolitis obliterans, associated with the Macklin effect (Figure 7).

However, several blood cultures remained negative. Moreover, the patient developed spontaneous subcutaneous emphysema and pneumomediastinum, associated with the Macklin effect. Importantly, bronchoscopy, gastroscopy, and thoracic CT and angio-CT did not reveal evidence of perforation or trauma. Combined treatment with meropenem, linezolid, ganciclovir, and voriconazol was initiated. Nevertheless, the patient’s condition worsened and he finally succumbed to respiratory failure after a 1-week period. An autopsy was refused by the relatives of the patient.

## 3. Discussion

PAMS can mimic SJS and TEN, which are rare, acute, and potentially life-threatening conditions, characterized by widespread epidermal necrolysis and sloughing. SJS and TEN are considered to originate from a common pathophysiology, and are classified based on the body surface area affected by detachment of the epidermis. The genetic background predisposing an individual to PAMS is different in patients from various races and countries. For example, HLA-Cw*14 may be the predisposing allele to PNP in patients descending from China, which is different from the predisposing allele in PAMS patients from France [12]. Drugs, in particular, antiepileptics and antibiotics, are the most common trigger factors of SJS/TEN. Infectious agents, including *Mycoplasma pneumonia*, have also been implicated [12,13,14]. Very rarely, vaccinations (e.g., those for influenza and rabies) have been associated with SJS/TEN [15,16]. However, we did not find any SJS/TEN cases in the literature that were associated with the herpes zoster, pneumococcus, and rubella vaccination, which the patient received one week prior to the onset of the initial symptoms. Even though vaccination-induced SJS was initially suspected, the long-term clinical course of this case suggests that the periodically recurring mucocutaneous skin manifestations were most likely not caused by the polyvalent vaccination in June 2020 [13,14,15,16].

Indeed, the subsequent detection of gastrointestinal infiltrates by follicular lymphoma helped to clarify the case. Follicular lymphoma is a type of indolent B-cell lymphoma, derived from follicular central cells and central blasts. The mechanism whereby this underlying disease causes PAMS remains unclear, but PAMS is always closely associated with the primary neoplasia. The initial symptoms of gastrointestinal follicular lymphoma manifestations include obstipation, abdominal pain, and intestinal bleeding. However, follicular lymphoma may also remain asymptomatic over a longer period [8,9,10,11]. With respect to the present case, we would like to argue that asymptomatic follicular lymphoma was the initial and persistent immunological trigger of the mucocutaneous lesions that arose, for the first time, in July 2020. The fact that there was no evidence for an autoimmune bullous disease by routine histopathology was probably the reason why direct and indirect immunofluorescence studies were not performed at that time.

The histologic findings in PAMS include epidermal acantholysis and clefting, the presence of necrotic keratinocytes, vacuolization of the basal layer, and lymphocyte exocytosis. Dyskeratosis, with suprabasal acantholysis, is a characteristic feature of PAMS in most cases. However, histopathologic features may vary with the particular morphology of the lesions, ranging from non-inflammatory bullous lesions to dense non-blistering lichenoid inflammatory infiltrates. Interface dermatitis, secondary to a T-cell-mediated autoimmune reaction, and subsequent infiltration into the epidermis suggest a strong cell-mediated cytotoxicity component in PAMS. Indeed, by immunohistochemistry, we observed a dense CD8+ lymphocytic infiltrate, associated with strong epidermotropism. Similarly, Kokubu et al. also observed a follicular lymphoma patient with TEN-like PAMS, showing an abundance of CD8+ cells infiltrating into the epidermis [17]. Importantly, we observed, for the first time, a chronic excess of peripheral cytotoxic lymphocytes, accompanied by significant inversion of the CD4/CD8 ratio, in a patient with PAMS. The chronically increased levels of CD8+ cells in the peripheral blood seem to indicate systemic cell-mediated autoimmunity, which was also reflected by the strong epidermotropic infiltrates of cytotoxic lymphocytes in the skin lesions. We can only speculate whether such an excess of CD8+ cells was also present in other organs, such as the esophagus and lungs. Importantly, the pathogenetic significance of cytotoxic cells in patients with PAMS or bronchiolitis obliterans has been previously emphasized by many authors [17,18,19,20,21,22,23,24].

Nguyen et al. [2] provided sophisticated experimental data showing that, in PAMS lesions, inducible nitric oxide species (iNOS)-positive keratinocytes were observed throughout the epidermis, and, thus, might have been targeted by CD8+ cytotoxic cells invading the epidermis. They also observed that most iNOS-positive keratinocytes were localized in the basal layer, which represents the predominant site of disruption of epidermal adhesion in PAMS [2]. Based on these data, the pathophysiology of PAMS differs significantly from classic pemphigus. Historically, paraneoplastic pemphigus was thought to represent an autoantibody-mediated condition exclusively caused by immune alterations and/or cross reactions with cancer-directed antibodies. However, mounting evidence indicates that CD8+ lymphocytes play a much more important role in the pathogenesis of PAMS than was initially assumed. Nguyen et al. have provided evidence that the mucocutaneous lesions in patients with PAMS occur as a result of both humoral and cell-mediated immune mechanisms [2]. The humoral autoimmunity responsible for the development of the condition may reflect, or even induce, a cell-mediated immune response that accounts for the localized inflammation, basal layer clefts, and sloughing of clustered epithelial cells [2]. Hence, a disease that is initially triggered by autoantibodies may result in autoantibody-dependent, or even -independent, CD8+-dominant cytotoxicity that may promote epitope spreading [25]. It is conceivable that autoreactive T-cell responses dominate, and may finally overtake, the disease progression of PAMS in some patients.

In the present case, it remains obscure whether humoral autoimmunity played a role at the beginning of the disease, since this was never determined. As would be expected, after the employment of anti-CD20 therapy, humoral autoimmune phenomena, i.e., circulating or deposited antibodies, were not detected. Interestingly, there are several reports on PAMS patients without detectable autoantibodies (e.g., desmoglein 1/3, envoplakin, and BP-230) [9,25,26,27,28]. Interestingly, most of these were patients who had received prior treatment with anti-CD20 antibodies for their lymphoproliferative disorder before the development of PAMS [25,26,27,28]. These observations strongly support the importance of autoreactive T-cell responses in PAMS. Interestingly, the frequency of PAMS diagnoses has declined, most likely due to the increased use of CD20-directed antibodies, which lead to negative results in direct and indirect immunofluorescence assays. A correct diagnosis is further complicated by the fact that PAMS, with its T-cell-mediated lichenoid phenotype, may mimic erythema multiforme, SJS, TEN, and graft-versus-host disease. Nonetheless, Lim et al. recently reported a patient with autoantibody-negative lichenoid PAMS, with no preceding anti-CD20 therapy, indicating that cell-mediated immune responses may predominate in some patients with PAMS [9]. Nevertheless, anti-CD20 therapy for the treatment of lymphomas, as well as immunosuppressive therapy for PAMS, carry a high risk for complications, due to infections. In the present case, endoscopic investigations showed severe sloughing of the esophageal epithelium. Furthermore, bronchoscopy revealed erythematous, highly vulnerable airway mucosa. Notably, the patient also developed spontaneous pneumomediastinum, associated with the Macklin effect, which can represent a complication of bronchiolitis obliterans [29,30,31]. Although generally occurring secondary to trauma, pneumomediastinum can also develop due to underlying lung conditions, such as asthma and bronchiolitis obliterans [29,30,31]. The Macklin effect can be frequently demonstrated in patients with spontaneous pneumomediastinum of non-traumatic respiratory causes, by multidetector-row CT. Hence, Sakai et al. [31] suggested that the Macklin effect, as revealed by CT scans, may be useful in differentiating respiratory from other causes of pneumomediastinum [31]. Accordingly, in this case, bronchoscopy, gastroscopy, and thoracic CTs did not reveal evidence of perforation or other trauma. Similar to another case of PAMS, reported by Odanit et al. [32], the patient discussed here very likely succumbed to his complicated bronchiolitis obliterans associated with co-infection by several pathogens.

Notably, Hata et al. [21] performed sophisticated experiments using a murine model. When mouse models of pemphigus vulgaris and PAMS were compared, the latter showed significantly higher mortality. Notably, intense CD4+ and CD8+ T-cell infiltrations were found in the peri-bronchial area in the lungs of PAMS mice, but not in pemphigus vulgaris mice. Moreover, it was discussed that the identification of T-cell-targeted antigens is more difficult than the targeting of IgGs, because T-cell receptors only recognize antigenic peptides when presented in the context of MHC I and II molecules [21]. Interestingly, their findings indicated that the ectopic expression of desmoglein 3 in the pulmonary epithelial cells was sufficient to recruit desmoglein-3-specific T cells. The T-cell infiltration observed in the lungs of PAMS mice consisted of a mixture of CD4+ and CD8+ cells. Hence, Hata et al. [21] concluded that a combination of CD4+ and CD8+ T cells is more efficient than each population alone to induce lung injury.

In the present case, the following findings indicate that cell-mediated immune responses were, indeed, the pathogenetic driver: (1) histology did not provide evidence for acantholysis; (2) an increase in peripheral blood CD8+ lymphocytes was already present at the beginning of the clinical symptoms; (3) immunohistology revealed an abundance of cytotoxic lymphocytes invading the epidermis; (4) during the whole course of the disease, the patient displaced a very low number of peripheral B lymphocytes; (5) anti-CD20 therapy had no effect on PAMS. Finally, the discovery that this case of PAMS was accompanied by a chronic elevation of peripheral blood CD8+ lymphocytes merits further investigation. Indeed, it is possible that two types of PAMS exist, one that is, at least initially, driven predominantly by humoral autoimmunity, and another that evolves into, or is even initiated by, cellular immunity as the pathogenic driver. In this context, it will be of great interest to determine whether elevated peripheral CD8+ lymphocytes serve as an indicator of cellular immunity-mediated PAMS.

## 4. Conclusions

This case report highlights the challenges in diagnosing PAMS, particularly in a case where the direct and indirect immunofluorescence assays were negative. Importantly, we observed, for the first time, a chronically increased number of CD8+ lymphocytes in the peripheral blood of a patient with PAMS, consistent with the systemic, autoreactive T-cell-driven processes that characterize this condition. The dense CD8+ infiltrate, with strong epidermotropism, observed in this case further supports the auto-aggressive, invasive role of cytotoxic lymphocytes in PAMS. Finally, this case informs future studies into the role of humoral versus cellular immunity in PAMS. Specifically, it will be of great interest to determine whether the presence of elevated peripheral blood CD8+ lymphocytes might indicate dependence on cellular immunity as the pathogenetic driver of PAMS.

## Figures and Tables

**Table 1:** Overview of laboratory findings from July 2020 to the beginning of November 2021 in a patient with paraneoplastic autoimmune multiorgan syndrome.

Parameter	18 July 2020	11 February 2021	9 September 2021	14 October 2021	29 October 2021 *	5 November 2021 **
Leukocytes (4600–9500/µL)	9770 ↑	10,330 ↑	15,950 ↑	4530 ↓	3030 ↓	3670 ↓
Erythrocytes (4.6–6.2 Mio/µL)	4.1 ↓	5.21	5.1	4.25 ↓	4.19 ↓	3.44 ↓
Hemoglobin (14–18 g/dL)	12.1 ↓	15.6	15	12.2 ↓	12.1 ↓	9.8 ↓
Thrombocytes (150,000–40,000/µL)	269,000	217,000	216,000	195,000	76,000 ↓	21,000 ↓
Lymphocytes (1000–4050/µL)	2240	3320	4090	330 ↓	250 ↓	140 ↓
Neutrophils (1800–7200/µL)	6410	5710	8530 ↑	2898	2550	3400
Eosinophils (40–360/µL)	30 ↓	250	1600 ↑	70	10 ↓	20↓
INR (0.8–1.1)	1.13 ↑	1.14 ↑	1.21 ↑	1.21 ↑	1.35 ↑	1.4 ↑
Lactate dehydrogenase (135–225 U/L)	184	164	141	208	220	254 ↑
GOT (10–50 U/L)	16	19	18	18	16	17
GPT (10–50 U/L)	26	15	23	18	16	13
Serum creatinine (0.7–1.2 mg/dL)	0.92	1.1	1.0	0.84	0.73	1.0
C-reactive protein (<5 mg/L)	13.9 ↑	<5	7.3 ↑	9.1 ↑	111.3 ↑	81.5 ↑
Procalcitonin (<0.5 ng/mL)	-	-	-	-	<2	0.68 ↑

* Admission to intensive care unit; ** one day before exitus letalis; GOT = glutamic oxaloacetic transaminase; GPT = glutamic pyruvic transaminase.

## Data Availability

Not applicable.
